# E3 ubiquitin ligase TRIM29 promotes pancreatic cancer growth and progression via stabilizing Yes-associated protein 1

**DOI:** 10.1186/s12967-021-03007-w

**Published:** 2021-08-05

**Authors:** Xueqiang Deng, Xiaowei Fu, Hong Teng, Lu Fang, Bo Liang, Rengui Zeng, Lian Chen, Yeqing Zou

**Affiliations:** 1 Department of Orthopedics, The Second Affiliated Hospital of Nanchang University, Nanchang, 330006 China; 2 Department of General Surgery, The Second Affiliated Hospital of Nanchang University, Nanchang, 330006 China; 3 Jiangxi Province Key Laboratory of Molecular Medicine, The Second Affiliated Hospital of Nanchang University, 1 Minde Road, Nanchang, 330006 Jiangxi China; 4 Department of Medical Ultrasonics, The First Affiliated Hospital of Nanchang University, Nanchang, 330006 Jiangxi China

**Keywords:** TRIM29, YAP1, Pancreatic cancer, Proliferation, Cell cycle, Cell apoptosis

## Abstract

**Background:**

Pancreatic cancer (PC) is one of the most fatal digestive system cancers. tripartite motif-29 (TRIM29) has been reported as oncogene in several human cancers. However, the precise role and underlying signal cascade of TRIM29 in PC progression remain unclear.

**Methods:**

Western blot, qRT-PCR and immunohistochemistry were used to analyze TRIM29 and Yes-associated protein 1 (YAP1) levels. CCK8 assays, EdU assays and flow cytometry were designed to explore the function and potential mechanism of TRIM29 and YAP1 in the proliferation of PC. Next, a nude mouse model of PC was established for validating the roles of TRIM29 and YAP1 in vivo. The relationship among TRIM29 and YAP1 was explored by co-immunoprecipitation and in vitro ubiquitination assay.

**Results:**

TRIM29 and YAP1 was significantly upregulated in PC patient samples, and TRIM29 expression was closely related to a malignant phenotype and poorer overall survival (OS) of PC patients. Functional assays revealed that TRIM29 knockdown suppresses cell growth, arrests cell cycle progression and promotes cell apoptosis of PC cells in vivo and in vitro. Furthermore, the rescue experiments demonstrated that TRIM29-induced proliferation is dependent on YAP1 in PC cells. Mechanistically, TRIM29 regulates YAP1 expression by directly binding to YAP1, and reduced its ubiquitination and degradation.

**Conclusion:**

Taken together, these results identify a novel mechanism used by PC growth, and provide insight regarding the role of TRIM29 in PC.

**Supplementary Information:**

The online version contains supplementary material available at 10.1186/s12967-021-03007-w.

## Background

Pancreatic cancer (PC) is a common malignant tumour in the digestive system and is characterised by a high malignancy, rapid growth, and a low early diagnosis rate [1, 2]. Therefore, the prognosis of patients with PC remains poor. Among the digestive tract malignant tumours, PC is one of the three major causes of death, with a 5-year survival rate lower than 5%. As most patients are diagnosed at an advanced or metastatic stage, the surgical resection rate is only 15% [3]. Currently, chemotherapy is one of the commonest treatment modalities for PC. However, studies have found that many patients develop chemotherapy resistance [4]. Before further progress is made regarding the current surgical methods, we need to find novel effective molecular targets to provide a better theoretical basis for a targeted treatment of PC.

TRIM29 (tripartite motif-29), also known as ATDC, is a member of the TRIM protein family. The TRIM family consists of more than 70 members. These proteins are characterised by a series of conserved domains, including B-box1, B-box2, ring, and RBCC domain motifs [5]. Trim family proteins are involved in many biological processes, including cell development, differentiation, apoptosis, and tumorigenesis [6–8]. TRIM29, located on chromosome 11q23, was initially considered to be an ectopic gene responsible for ataxia telangiectasia. It is involved in cell growth and plays a role in several processes such as immune inflammatory mediation, cell signal transduction, protein translocation, cell apoptosis, and cell cycle regulation [9,10]. Studies have also shown that its expression is upregulated in gastric cancer [11], lung cancer [12], and osteosarcoma [13]. Owing to its E3 ubiquitin ligase activity, in many cancers, TRIM29 contributes to the ubiquitination of target proteins and tags them for degradation; for example, TRIM29 promotes the progression of lung cancer by stabilising β-catenin [14]. However, the precise role and underlying signal cascade of TRIM29 in PC progression remain unclear.

Recently, several studies have confirmed that Hippo/YAP is a highly conserved growth regulatory signalling pathway that plays a key role in cell proliferation and apoptosis [15]. The kinase cascade is the key to signal transduction. YAP1, a key protein downstream of the Hippo signalling pathway, has been confirmed to play an important role in the malignant progression of tumours [16]. However, the regulation mechanism of PC requires further study.

In the present study, we aimed to elucidate the role of TRIM29 in the PC progression. We also investigated the mechanism underlying the effects of YAP1 in PC. Taken together, our data suggests new potential prognostic and therapeutic targets for PC.

## Materials and methods

### Human tissue specimens

Informed consent was obtained from the patients. We collected 126 fresh PC samples and corresponding adjacent tissues from 78 male patients and 48 female patients treated at the Second Affiliated Hospital of Nanchang University from January 2013 to December 2018. All samples were frozen and stored at − 80 °C until required. The patients enrolled in our study received neither chemotherapy nor radiotherapy prior to surgery. This study was approved by the ethics and research committees of the Second Affiliated Hospital of Nanchang University and was conducted in accordance with the Declaration of Helsinki Principles.

### Cell lines and antibodies

Four human PC cell lines (SW1900, PANC-1, AsPC-1, and BxPC-3) and a normal human pancreatic ductal epithelial cell line HPDE6-C7, were obtained from the American Type Culture Collection (Rockville, MD, USA). PC cell lines were routinely maintained in Dulbecco’s modified Eagle’s medium supplemented with 10% fetal bovine serum (FBS; Gibco, Grand Island, NY, USA) at 37 °C in a humidified incubator set at 5% CO_2_. HPDE6-C7 cells were cultured in keratinocyte serum-free medium (Invitrogen, Carlsbad, CA, USA) supplemented with 10% FBS and antibiotics in a humidified incubator set at 37 °C and 5% CO_2_ atmosphere. The primary antibody against TRIM29 (Abcam, ab108627) was used (1:1,000 dilution) for western blotting and for immunohistochemistry (1:200 dilution). The primary antibody against YAP1 (Abcam, ab205270) was used (1:1,000 dilution) for western blotting and for immunohistochemistry (1:150 dilution). The primary antibodies against Ub (Abcam, ab134953), PCNA (Abcam, ab92552), Cyclin D1 (Abcam, ab40754), BCL2 (Abcam, ab32124), Bax (Abcam, ab32503), and Caspase 3 (Abcam, ab32351) were used at 1:1,000 dilution for the western blot, and the primary antibody against GAPDH (Abcam, ab181602) was used at a 1:3,000 dilution for western blotting (Additional files 1, 2 and 3).

### Quantitative real-time PCR (qRT-PCR)

Total RNA was extracted from cells and tissues using TRIzol Reagent (Thermo Fisher Scientific, Waltham, USA). We use a 1% agarose gel electrophoresis experiment to verify the integrity of our extracted RNA; and use a nucleic acid quantitative analyser to determine the concentration and purity of RNA. cDNA was synthesised by reverse transcription using the PrimeScript RT Reagent Kit (Takara, Dalian, China). Then, quantitative PCR was performed using SYBR Premix Ex Taq (Takara, Dalian, China), following the manufacturer’s instructions. GAPDH were regarded as the internal control, and the relative gene expression was determined using the 2^−ΔΔCT^ method. All experiments were repeated three times. All primers were designed by Ribbo (Guangzhou, China) and their sequences are as follows: TRIM-29, forward: 5′-TGCGAGCTGCATCTCAAGC-3′, reverse: 5′-GGTGCTATGATTCTTGTGCTCC-3′; YAP1, forward: 5′-GCAACTCCAACCAGCAGCAACA-3′, reverse: 5′-CGCAGCCTCTCCTTCTCCATCTG-3′; GAPDH, forward: 5′-AGCCTCAAGATCATCAGCAATG-3′, reverse: 5′-CCATCACGCCACAGTTTCC-3′.

### Western blot

Total cellular protein was extracted using RIPA buffer (Beyotime, Guangzhou, China), and the tissue protein was extracted using the extraction reagent. BCA assay was performed to determine the protein concentration. Total 40 μg protein from each sample was subjected to SDS-PAGE and transferred to PVDF membranes. After blocking the membranes with skim milk, they were incubated overnight with the corresponding antibody at 4 °C. After washing the PVDF membrane with TBST, the membranes were incubated with the corresponding secondary antibody for 1.5 h at room temperature. Finally, the proteins were visualized with enhanced chemiluminescence reagents (Millipore).

### Immunohistochemistry (IHC)

Fresh clinical tissue samples stored at − 80 °C were fixed in 4% neutral paraformaldehyde as soon as possible, embedded in paraffin, and sliced in 4-μm-thick section. The samples were dried overnight in an oven. The samples were dewaxed and hydrated. A 1% Triton X-100 solution was added to the samples followed by osmotic treatment. Samples were blocked with 5% BSA for 30 min, treated with the corresponding primary and secondary antibodies, and stained with DAPI.

### Cell growth assay

For determining the logarithmic growth, PC cells were treated according to established experimental groups. In each treatment group, cells (1 × 10^4^ cells/well) were plated in a 96-well plate and cultured for 24 h. For the CCK8 assays, 10 μL of CCK8 reagent was added into each well and incubated for 10 min. The OD values were determined at 405 nm. For the EdU assay, PC cells were incubated with 5-ethynyl-20-deoxyuridine (EdU; RiboBio, Guangzhou, China) for 5 h and were subsequently processed according to the manufacturer’s instructions.

### Flow cytometry

PC cells were treated according to the methods mentioned above. After trypsin digestion, the cells were counted and 10 × 10^5^ cells were placed in the flow tube, washed twice with PBS, and incubated in the dark for 30 min with PI and Annevin-V reagent. The apoptosis rate was detected using flow cytometry, and the average value was obtained from three independent experiments performed for each group.

### *Co-immunoprecipitation (Co-IP) and *in vitro* ubiquitination assay*

Co-IP assays were performed as previously described [17]. For the in vivo ubiquitination assay, PC cells subjected to TRIM29 knockdown or overexpression were exposed to MG132 treatment for 6 h before harvesting. The cell lysates were prepared and immunoprecipitated with anti-YAP1 antibody. The ubiquitination level of YAP1 was assessed using an anti-Ub antibody.

### Tumorigenicity assay

PC cells (1 × 10^6^ in 100 mL of PBS) were injected subcutaneously into the flanks of nude mice (male BALB/c-nu/nu, 6–8 weeks old). Tumour formation in nude mice was monitored, and the tumour volume was measured every 5 days. Tumours were harvested and individually weighed after the mice were anaesthetised. The data are presented as tumour weight (mean ± SD). The animal experiments were performed in accordance with the experimental animal use guidelines of the National Institutes of Health and approved by the Ethics Committee for Animal Experiments of the Second Affiliated Hospital of Nanchang University.

### Gene expression profiling interactive analysis (GEPIA) database

GEPIA (http://gepia.cancer-pku.cn/), a multifunctional molecular analysis platform based on TCGA data and GETx data containing 179 pancreatic cancer samples and 171 normal samples altogether, was employed to compare the mRNA expressions of TRIM29 and YAP1 between pancreatic cancer and adjacent normal tissues. *P*-values were generated using the Student’s t-test. *P*-value < 0.05 signified differential expression of a gene in the two sets of tissues.

### Statistical analysis

The data obtained in the current study was acquired from experiments run in triplicate and is expressed as the mean ± SD. The data was analysed using GraphPad Prism 7 and SPSS 26.0 software. Differences between two groups were analysed using *t*-tests or Student’s *t*-tests. Multiple group comparisons were performed using one-way analysis of variance (ANOVA) followed by Student–Newman–Keuls test as the post hoc test. Spearman’s correlation test was used for correlation analyses. Results with *p* < 0.05 were considered statistically significant.

## Results

### TRIM29 expression is upregulated and correlated with survival in PC

**Fig. 1:**
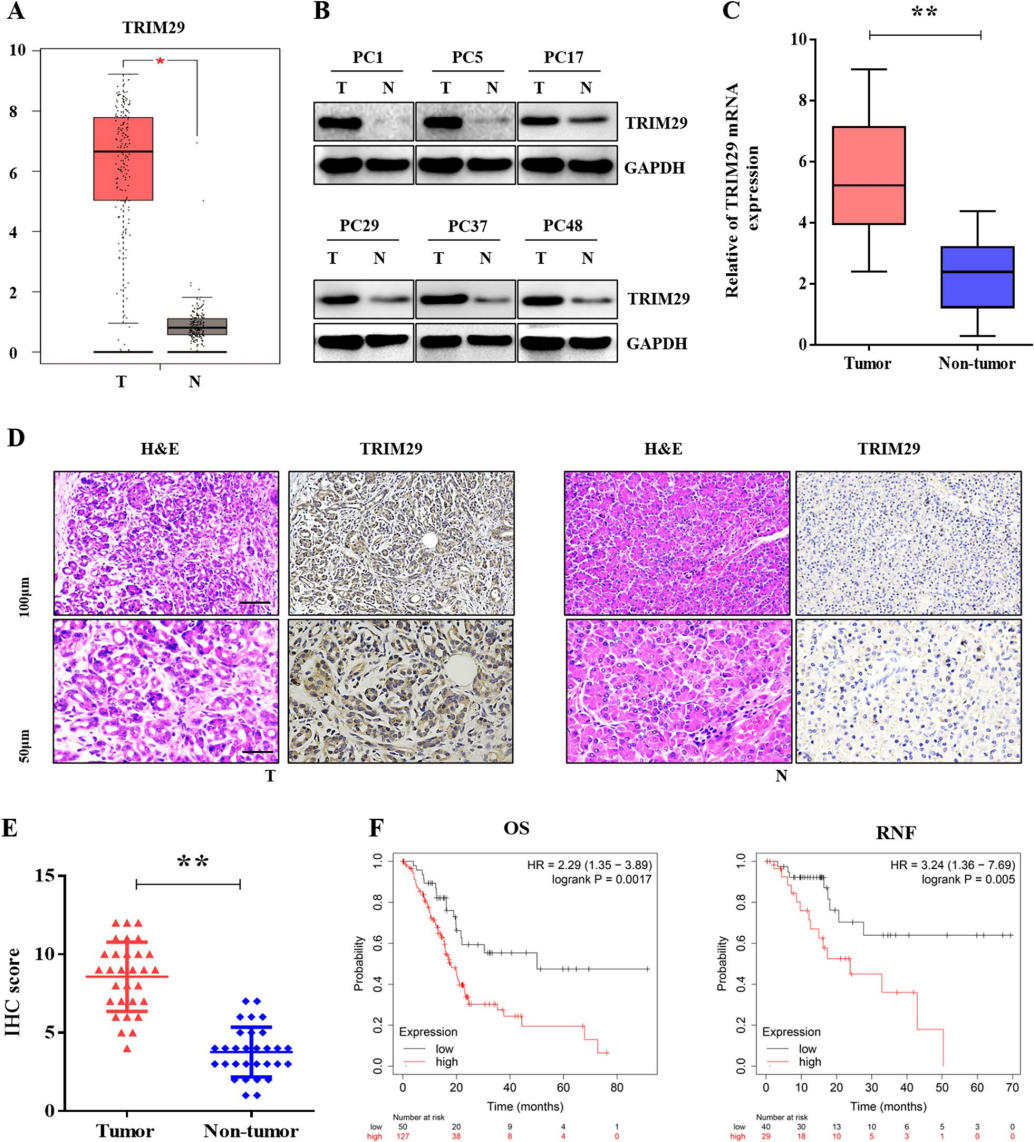
TRIM29 expression was upregulated and correlated with survival in pancreatic cancer. **A** GEPIA database analysis showed that the expression of TRIM29 was significantly increased in pancreatic cancer (PC) (*p < 0.05, T means tumour; N means normal). **B** and **C**, Determination of TRIM29 protein and mRNA levels in PC tissues and paired non-tumour tissues using western blotting and qRT-PCR. GAPDH was used as an internal control (** *p* < 0.01, N = Normal, T = Tumour). Representative images (**D**) and quantification (**E**) of TRIM29 IHC staining in 126 paired PC and non-cancer tissues collected in our center. A high TRIM29 expression was observed in 66.04% (77/126) (** *p* < 0.01) of samples. F, Kaplan–Meier survival curves revealed that the patients with high levels of TRIM29 expression exhibited a shorter overall and disease-free survival than those with lower levels

To detect the expression level of TRIM29 in PC tissues, we initially analysed TRIM29 expression using GEPIA database software. As shown in Fig. 1A, the expression of TRIM29 was significantly higher in PC tissues than in the adjacent non-tumour tissues. Next, we analysed the expression of TRIM29 using western blot and qRT-PCR in PC tissues. Among the 50 specimens of tumour tissues, the protein expression of TRIM29 was significantly increased in 37 specimens. The difference was not statistically significant in 13 cases, and the representative western blots of six tissue samples are shown in Fig. 1B. The results of qRT-PCR indicated that the TRIM29 mRNA level in PC tissues was approximately two-fold higher than that in the corresponding adjacent tissues (Fig. 1C). We further analysed the expression of TRIM29 in PC tissues using IHC. As shown in Fig. 1D, E, TRIM29 reactivity was observed in 66.04% (77/126) of the PC specimens collected at our center, while a negative staining was observed in the corresponding adjacent non-tumour tissues.

Next, we analysed the relationship between TRIM29 expression and clinicopathological parameters in PC patients. As shown in Table 1, TRIM29 overexpression was closely correlated with tumour size and lymph node metastasis, but did not correlate significantly with age, sex, or tumour location. Furthermore, Kaplan–Meier survival curves (Fig. 1F) revealed that patients with high levels of TRIM29 expression exhibited a shorter OS than those with lower levels of TRIM29. Taken together, these data suggest that the TRIM29 expression is significantly upregulated in PC tissues and affects PC progression.

**Table 1:** Correlation between TRIM29 expression and clinicopathologic features in 126 patients with pancreatic ductal adenocarcinoma

Characteristics	Total	TRIM29 expression		P value
		Low (n = 49)	High (n = 77)	
Age (years)				*P* = 0.951
≤ 60	51	20	31	
> 60	75	29	46	
Gender				*P* = 0.531
Male	78	32	46	
Female	48	17	31	
Tumor grade				*P* = 0.328
Poorly	46	20	26	
Moderately	54	17	37	
Well	26	12	14	
Tumor stage				*P* = 0.903
I–II	60	23	37	
I–II–IV	66	26	40	
Tumor size				***P* = *0.003***
≤ 20 mm	40	23	17	
> 20 mm	86	26	60	
Lymph node metastasis				***P* = *0.001***
Positive	75	20	55	
Negative	51	29	22	
Vascular invasion				*P* = *0.568*
Positive	68	28	40	
Negative	58	21	37	
Perineural invasion				*P* = *0.460*
Positive	72	30	42	
Negative	54	19	35	

### *Knockdown of TRIM29 expression suppressed the growth of PC cells *in vitro* and *in vivo

**Fig. 2:**
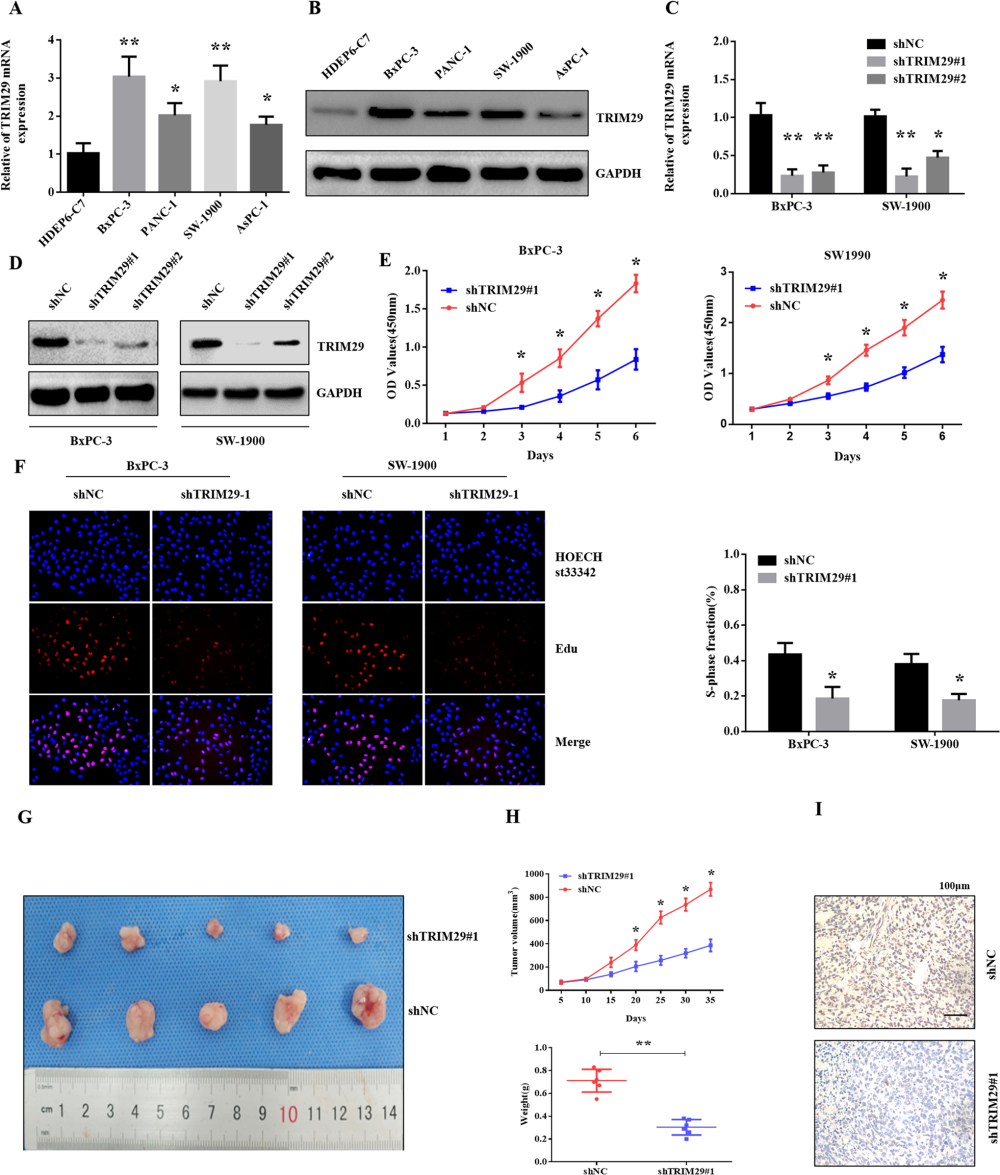
Knockdown of TRIM29 expression suppressed the growth of pancreatic cancer cells in vitro and in vivo*.* **A** and **B**, qRT-PCR and western blot analysis of TRIM29 expression in pancreatic cancer (PC) cell lines (SW1900, PANC-1, AsPC-1, and BxPC-3) and a normal human pancreatic ductal epithelial cell line HPDE6-C7 (*p < 0.05, **p < 0.01). **C** and **D**, after transfection with shTRIM29 plasmids, qRT-PCR and western blotting showed that the level of TRIM29 was reduced (**p < 0.01). E and F, CCK8 and EdU assays showed that shTRIM29 cell proliferation was inhibited (*p < 0.05). **G** and **H**, BxPC-3/shTRIM29 and BxPC-3/shNC cells were injected subcutaneously into nude mice, and the tumour volumes were measured on the indicated days. At the end of the experiment, the tumours were dissected, photographed, and weighed (n = 5, * *p* < 0.05). **I** IHC assay detected the expression of BxPC-3/shTRIM29 and BxPC-3/shNC

To clarify the expression of TRIM29 in PC cells, we analysed the expression of TRIM29 in different PC cell lines. The results showed that the expression of TRIM29 in SW1900, PANC-1, AsPC-1, and BxPC-3 cells was significantly higher than that in the normal human pancreatic ductal epithelial cell line HPDE6-C7 (Fig. 2A, B). Further, we used two shRNA (shRNATRIM29#1 and shRNATRIM29#2) to knockdown TRIM29 in BxPC-3 and SW-1900 cell lines, and our results shown that shRNATRIM29#1 have stronger effect in PC cell (Fig. 2C, D). We performed CCK8 and EdU assays to analyse the effect of TRIM29 expression on the growth of BxPC-3 and SW1900 cells. Our results showed that TRIM29 knockdown significantly inhibited cell viability compared with the control group (Fig. 2E, F).

To further investigate the effect of TRIM29 expression on PC in vivo, we performed studies using a nude mouse subcutaneous xenograft model. We observed that xenografts derived from TRIM29-knockdown BxPC-3 cell grew at a significantly slower rate compared with their respective controls (Fig. 2G). Similarly, the average tumour weight in mice bearing BxPC-3/shTRIM29 cells was significantly lower (Fig. 2H). Finally, using IHC assays, we confirmed that TRIM29 expression was significantly decreased in BxPC-3/shTRIM29 cells (Fig. 2I). Collectively, these data indicate that TRIM29 contributes to PC growth, both in vitro and in vivo.

### Knockdown of TRIM29 expression arrests cell cycle progression and promotes cell apoptosis

**Fig. 3:**
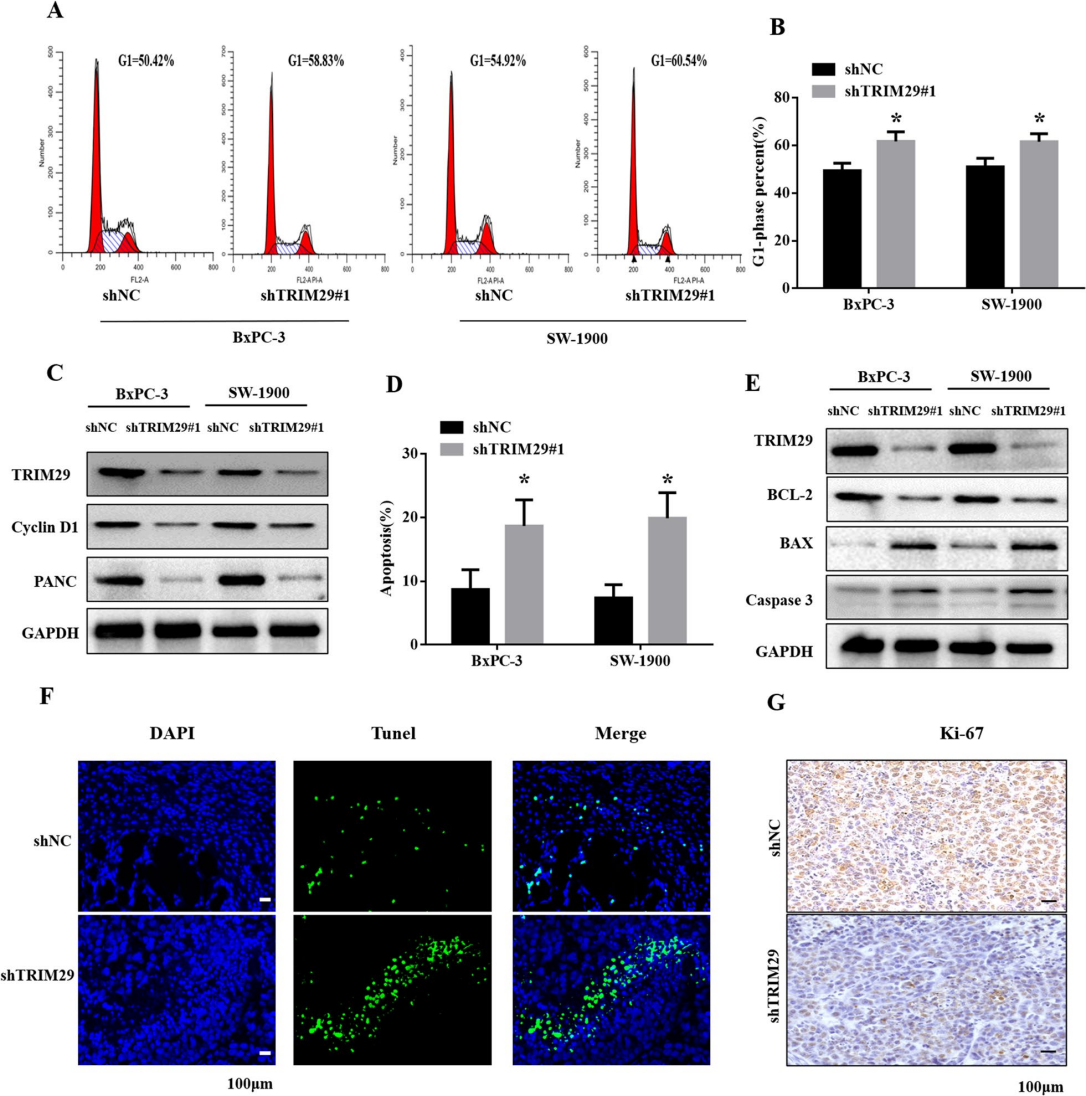
Knockdown of TRIM29 induced cell cycle progression and promoted cell apoptosis. **A** and **B**, after down-regulating TRIM29 expression in pancreatic cancer (PC) cells, the cell cycle was arrested in the G1 phase (*p < 0.05). **C** western blot results showed that the expression levels of cyclins Cyclin-D1 and PCNA decreased. **D** apoptosis experiments confirmed that the proportion of phase apoptosis was significantly increased in shTRIM29 cells (*p < 0.05). **E** The expression of BAX and caspase 3 was increased in shTRIM29 cells, while that of BCL-2 was reduced. **F** TUNEL staining images for apoptosis in the two tumour xenograft groups. G, IHC for Ki-67 in the two tumour xenograft groups

To further investigate the potential mechanisms of TRIM29 on the growth of PC cells, we performed flow cytometry to analyse the cell cycle. The results indicated that the knockdown of TRIM29 expression significantly arrested the PC cells in the G1 phase (Fig. 3A, B). Similarly, the western blotting results showed that TRIM29 knockdown led to decreased levels of cyclin D1 and PCNA (Fig. 3C). Furthermore, we assessed the effect of TRIM29 on the apoptosis of PC cells. The results showed a significant increase in the apoptosis rate in shTRIM29 cells (Fig. 3D). Similarly, the results of western blot analysis showed that TRIM29 knockdown led to decreased BCL-2 levels and increased Bax and Caspase 3 levels (Fig. 3E). TUNEL analysis further confirmed that TRIM29 knockdown increased the number of apoptotic cells in xenograft tumours (Fig. 3F). IHC assay also confirmed that Ki-67 was significantly decreased in shTRIM29 cell-derived xenografts (Fig. 3G). Taken together, these results showed that TRIM29 knockdown can arrest the cell cycle at the G1-phase and promote cell apoptosis of PC cells.

### TRIM29 positively regulates the expression of YAP1 protein

**Fig. 4:**
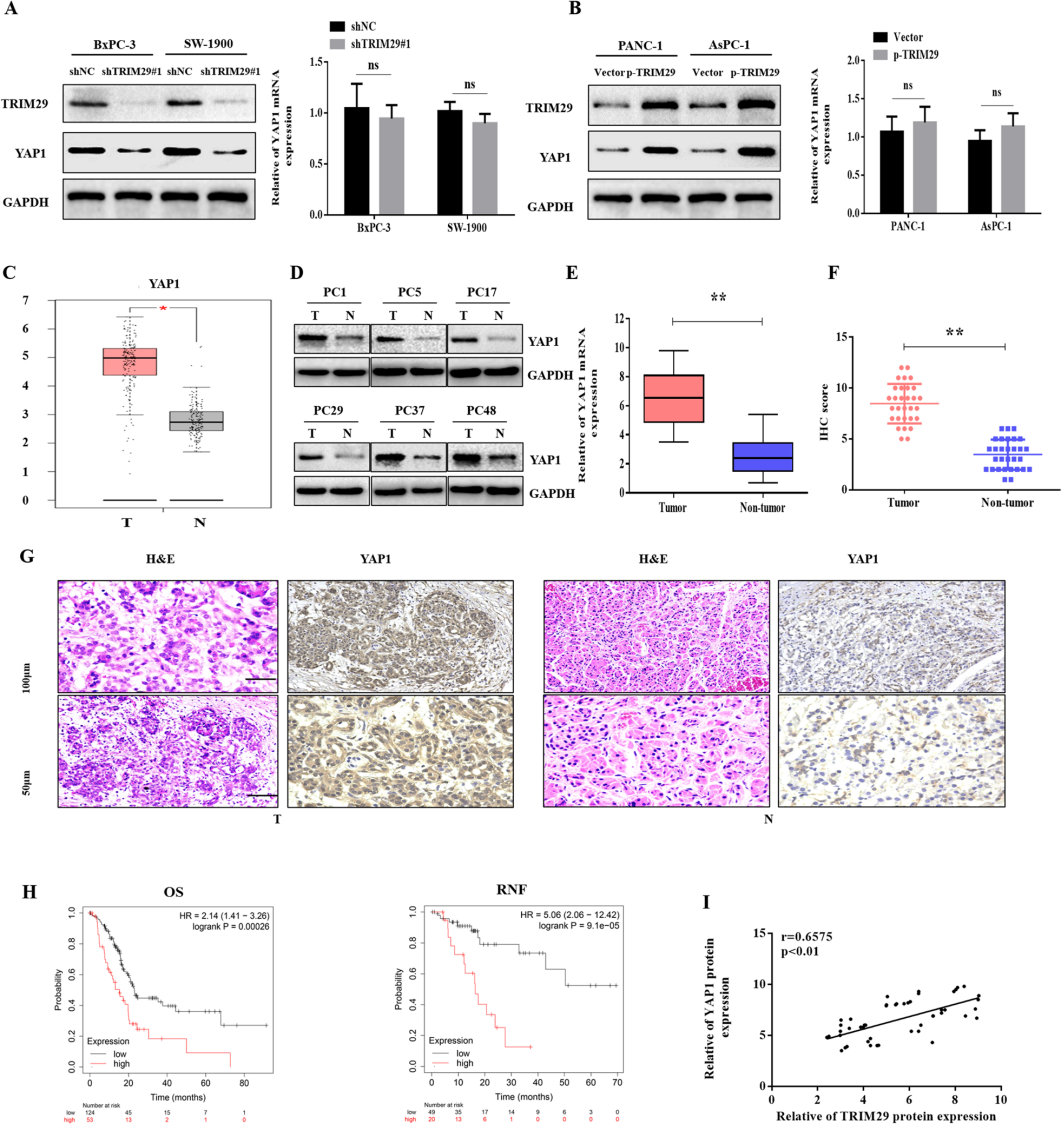
TRIM29 positively regulated YAP1 protein level. **A** and **B**, in response to upregulating or downregulating TRIM29 expression in pancreatic cancer (PC) cells, the expression level of YAP1 protein also changed, but its mRNA level changed insignificantly (ns means not significantly). **C** GEPIA database analysis showed that the expression of YAP1 was significantly increased in PC (*p < 0.05, T means tumour; N means normal). **D** and **E**, Determination of YAP1 protein and mRNA levels in PC tissues and paired non-tumour tissues using western blotting and qRT-PCR. GAPDH was used as an internal control (** *p* < 0.01, N = Normal, T = Tumour). Representative images (**G**) and quantification (**F**) of YAP1 IHC staining in 126 paired PC and non-cancer tissues. A high YAP1 expression was observed in 62.70% (79/126) (** *p* < 0.01) of samples. H, Kaplan–Meier survival curves revealed that PC patients with high levels of TRIM29 expression exhibited a shorter overall and disease-free survival than those with lower levels. **I** Scatter plots of TRIM29 and YAP1 expression in PC tissues (*p < 0.05, r = 0.6575)

Previous studies have shown that the Hippo/YAP signalling pathway plays an important role in tumour progression, especially in progression of malignant PC [18], but the specific molecular mechanism has not been fully elucidated. Interestingly, our results showed that the protein level of YAP1 decreased when TRIM29 was downregulated in PC cells. In contrast, when TRIM29 was overexpressed, the protein level of YAP1 also increased, however, its mRNA level remained unchanged (Fig. 4A, B). To further illustrate the role and mechanism of YAP1 in PC, we analysed the expression of YAP1 in PC using the GEPIA database software, and the results showed that the expression of YAP1 in PC tissues was significantly higher than in adjacent tissues (Fig. 4C). Results of western blot, qRT-PCR, and IHC assays also confirmed that YAP1 was overexpressed in PC tissues (Fig. 4D–G). Kaplan–Meier survival curves (Fig. 4H) revealed that patients with high levels of YAP1 expression exhibited a shorter OS than those with lower levels of YAP1. Furthermore, the scatter plots showed that TRIM29 and YAP1 expression levels were positively correlated in the PC tissues (Fig. 4I). Taken together, these findings suggest that TRIM29 promoted the expression of YAP1, and thus, promoted the proliferation of PC cells.

### YAP1 mediates TRIM29-induced proliferation in PC cells

**Fig. 5:**
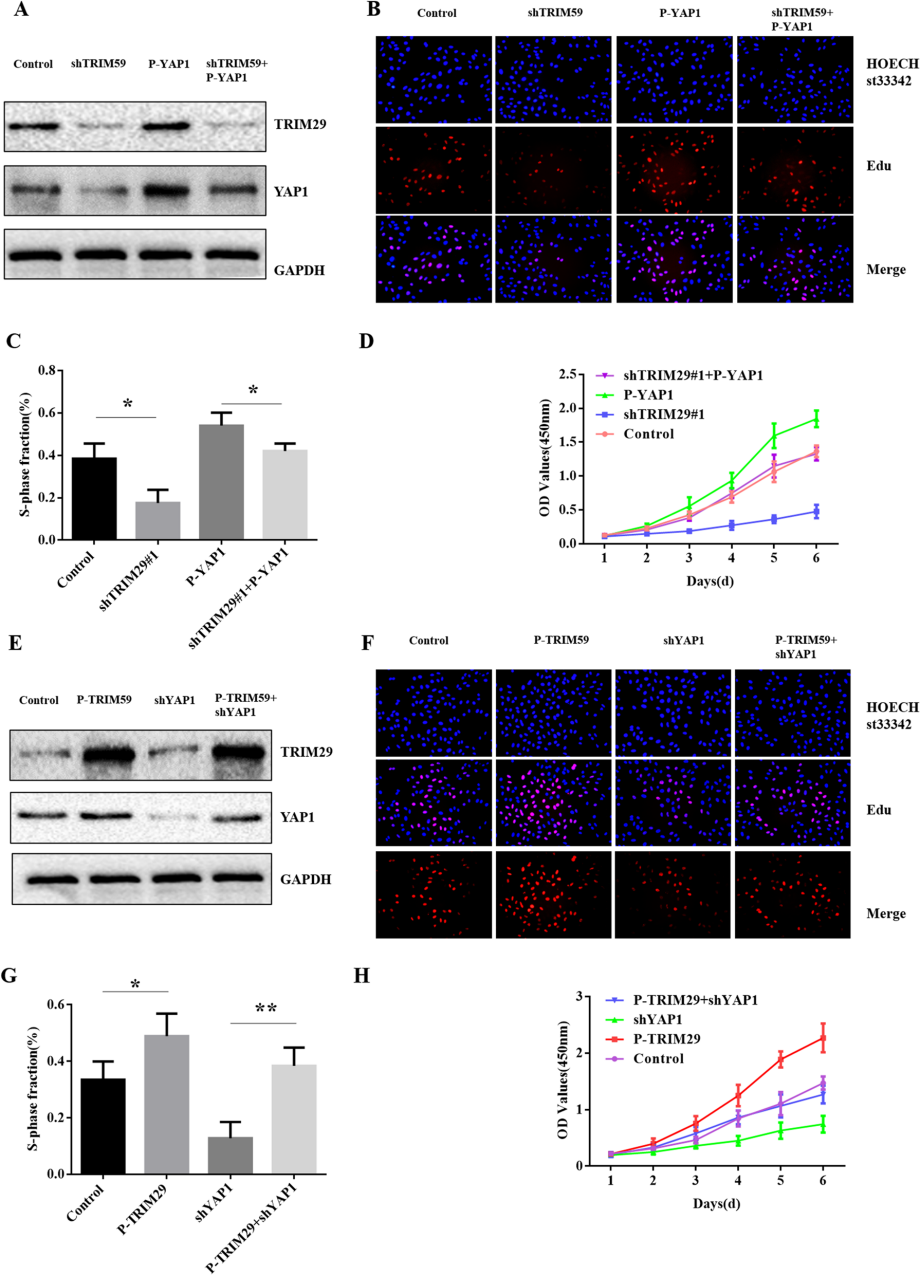
YAP1 mediated TRIM29-induced proliferation in pancreatic cancer cells. **A** western blot showing TRIM29 and YAP1 expression in BxPC-3 cells stably transfected with shTRIM29 in the presence or absence of P-YAP1. **B**–**D**, EdU and CCK8 assays showed that the reduced proliferation induced by TRIM29 knockdown in BxPC-3 cells was partially abolished by the introduction of P-YAP1 (*p < 0.05). **E** western blot analysis of TRIM29 and YAP1 protein expression in PANC-1 cells stably transfected with P-TRIM29 in the presence or absence of shYAP1. **F**–**H**, CCK8 and EdU assays showed that the increased proliferation induced by TRIM29 overexpression in PANC-1 cells was partially abolished by the introduction of shTRIM29

As discussed abo*ve,* YAP1 was determined to be a downstream gene of TRIM29. Next, we questioned whether YAP1 is a mediator of TRIM29-induced proliferation in PC cells. First, we upregulated the expression of YAP1 in TRIM29-knockdown BxPC-3 cells, and the upregulating efficacy was confirmed (Fig. 5A). CCK8 and EdU assays showed that the reduced proliferation induced by TRIM29 knockdown in BxPC-3 cells was partially abolished by the introduction of P-YAP1 (Fig. 5B–D). Next, we silenced the expression of YAP1 in TRIM29-upregulated SW-1900 cells. The silencing efficacy was confirmed (Fig. 5E). CCK8 and EdU assays showed that an increased proliferation promoted by TRIM29 upregulation in PANC-1 cells was partially abolished by the introduction of shYAP1 (Fig. 5F–H). Taken together, these findings suggest that YAP1 mediates TRIM29-induced proliferation in PC cells.

### TRIM29 stabilises YAP1 by regulating YAP1 ubiquitination in PC cells

**Fig. 6:**
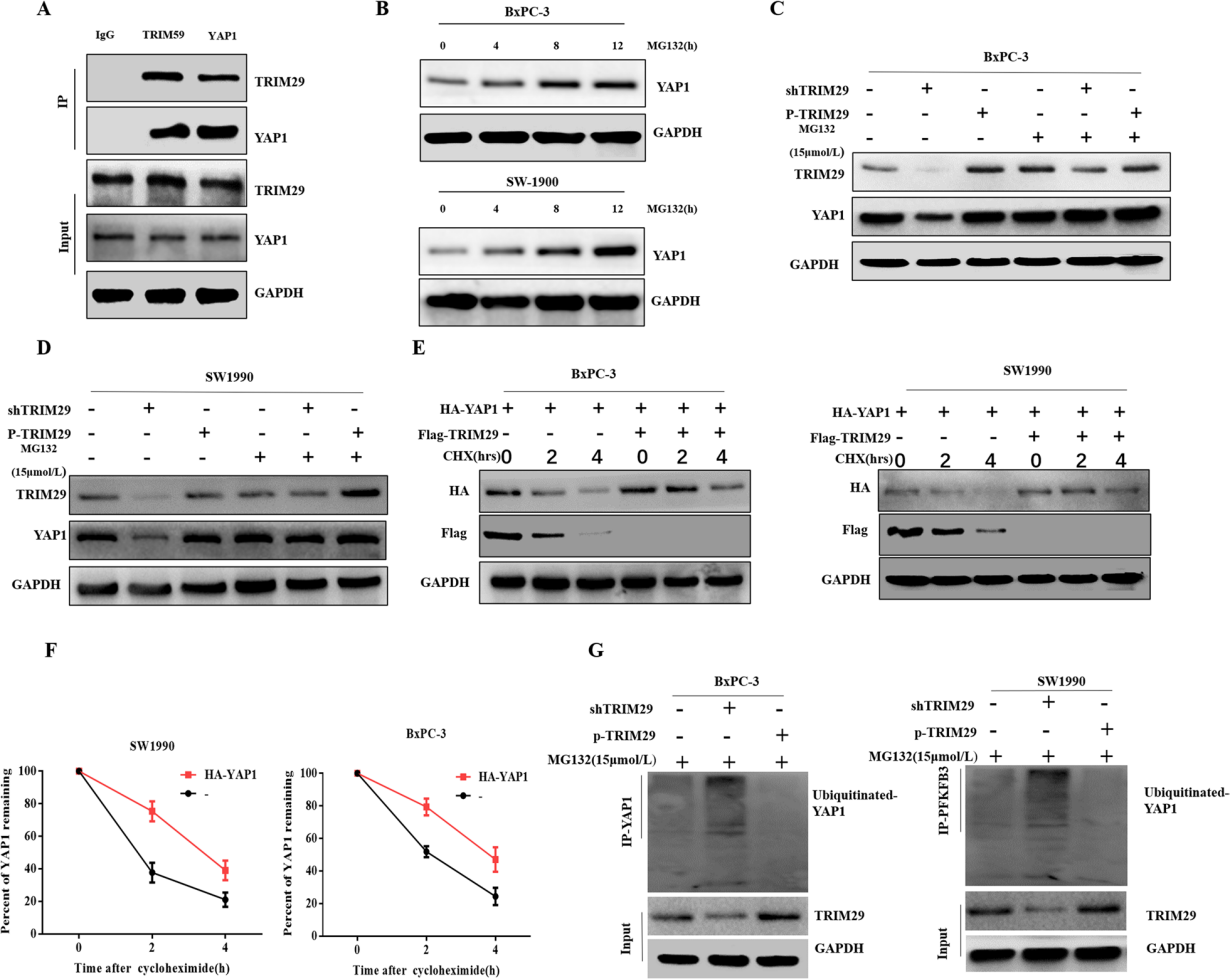
TRIM29 stabilized YAP1 by regulating its ubiquitination in pancreatic cancer cells. **A** co-IP results showed that TRIM29 and YAP1 interacted directly. **B** The expression level of YAP1 increased with time in pancreatic cancer (PC) cells treated with proteasome inhibitor MG132. **C** and **D** TRIM29 did not affect YAP1 expression level, as assessed in PC cells transfected with shTRIM29/P-TRIM29 plasmid and treated with MG132. **E** and **F** Detection of YAP1 degradation using anti-HA and anti-Flag antibodies in BxPC-3 and SW1990 cells transfected with the HA-YAP1 expression plasmid, with or without the Flag-TRIM29 plasmid, and treated with cycloheximide for the indicated times. **G** Lysates from PC cells transduced with shTRIM29/P-TRIM29 were treated with MG132 (15 µM) for 4 h, collected, immunoprecipitated using an anti-ubiquitin (Ub) antibody, and then immunoblotted using a YAP1 antibody

To further clarify the specific mechanism by which TRIM29 regulates YAP1, we first confirmed the direct binding of TRIM29 and YAP1 through immunoprecipitation (Fig. 6A). Studies have confirmed that TRIM29 can stabilise the degradation process of substrate proteins, and our previous studies also confirmed that YAP1 can be transformed from degradation [19]. Interestingly, our results confirmed that TRIM29 affected the expression level of YAP1 protein, although its mRNA expression did not change significantly. Therefore, we speculated that TRIM29 may affect YAP1 ubiquitination to stabilise its expression. To test this hypothesis, we treated PC cells with proteasome inhibitor MG132 (15 µM), and the results showed a significant accumulation of endogenous YAP1 protein in treated cells (Fig. 6B). This result demonstrates that YAP1 is also degraded by UPS in PC cells.

Next, we studied whether TRIM29 is involved in regulating the degradation process of YAP1. We added the proteasome inhibitor MG132 to shTRIM29 and P-TRIM29 PC cells. The results showed that TRIM29 expression had no significant impact on YAP1 level when PC cells were treated with MG132 (Fig. 6C, D). In addition, degradation kinetics experiments showed that the half-life of YAP1 was significantly longer in TRIM29-overexpressing PC cells than that in control cells (Fig. 6E, F). These results indicated that TRIM29 regulated the degradation of YAP1. Finally, to clarify the mechanism of YAP regulation by TRIM29, we treated PC cells with MG132 after transfecting with shTRIM29 and P-TRIM29 plasmids. Immunoprecipitation experiments using an anti-YAP1 antibody showed that TRIM29 knockdown and overexpression increased and reduced the level of YAP ubiquitination, respectively (Fig. 6G). Collectively, these results suggest that TRIM29 stabilises YAP1 by regulating its ubiquitination.

## Discussion

PC is a highly malignant neoplasm with poor prognosis. Due to the majority of patients are diagnosed at a later or metastatic stage, only no more than 20% of pancreatic cancer patients are resectable [20,21]. Targeted tumour therapy and neoadjuvant chemotherapy play important roles in inhibiting the progression of PC. The malignant growth of PC is one of the main reasons for the failure of surgical treatment. The malignant growth of tumours is regulated by multiple factors that act in multiple steps. Its occurrence and development involve the activation and inactivation of a number of oncogenes and tumour suppressor genes. Therefore, understanding the mechanisms underlying the malignant growth of PC will provide new insights for its better clinical management in future into this disease. Here, we demonstrated that a high expression of TRIM29 is predictive of a poor prognosis in PC and that TRIM29 plays an important role in PC progression.

The Tripartite motif-containing protein (TRIM) family is also called the RBCC family. It is a family of proteins with a relatively conservative structure that have evolved rapidly. Due to the unique structure of its constituents, research on the functions of the TRIM family members has received increasing attention in recent years [22,23]. TRIM29 is a new member of the TRIM family. Studies have confirmed that it is involved in important biological processes, such as cell proliferation, differentiation, apoptosis, and virus infection [24]. Several studies have shown that TRIM29 expression is significantly upregulated in a variety of tumours, such as colorectal cancer [25], gastric cancer [26], and lung cancer [27], and that the high expression of TRIM29 is closely related to its prognosis. However, in some tumours, especially in prostate cancer [28], TRIM29 is expressed at low levels. Our results showed that the TRIM29 expression was detected in the tumours obtained from patients with PC, compared with the corresponding non-tumour tissues. It is important to note that the high expression level of TRIM29 is related to the tumour size, lymph node metastasis, and a shorter overall survival in PC patients. Further studies have found that TRIM29 can affect the growth of PC cells in vivo and in vitro. Therefore, collectively, these findings suggest that TRIM29 may be a novel indicator of a poor prognosis in PC and may function as an oncogene in pancreatic cancer progression.

The malignant growth of tumours is a complex process involving multiple genes. To clarify the specific mechanism by which TRIM29 regulates PC growth, we focused on studying the downstream protein YAP1 of the Hippo signalling pathway. Studies have shown that YAP1 acts as a proto-oncogene in many tumours, including breast cancer [29]. It is abnormally overexpressed in colorectal cancer and osteosarcoma, and is also widely involved in the biological processes of tumours [30]. Interestingly, our study also confirmed that YAP1 is overexpressed in pancreatic cancer and the prognosis of PC patients with a high YAP1 expression is poor. Another study reported that the expression of TRIM29 and YAP1 is positively correlated in PC [14]. In PC cells, YAP1 expression was downregulated in response to decreased TRIM29 expression; on the contrary, the expression of YAP1 increased when TRIM29 was overexpressed. Further, downregulating the expression of YAP1 in PC cells can attenuate the effect of TRIM29 overexpression on the growth of PC cells. The overexpression of YAP1 can restore the inhibitory effect of TRIM29 downregulation on the growth of PC cells. Studies have shown that the E3 ubiquitin ligase TRIM29 can stabilise the degradation process of substrate proteins. Our previous studies have shown that YAP1 can be degraded by ubiquitination and the results of the present study show that TRIM29 does not affect YAP1 mRNA expression. Thus, we speculate that TRIM29 stabilised the expression of YAP1, which may affect the ubiquitination of latter. Moreover, our results showed that TRIM29 is directly bound to YAP1 in PC cells and downregulating the expression of TRIM29 in PC significantly promoted YAP1 ubiquitination. In contrast, TRIM29 overexpression reduced ubiquitination. Thus, TRIM29 can increase the half-life of YAP1.

## Conclusions

**Fig. 7:**
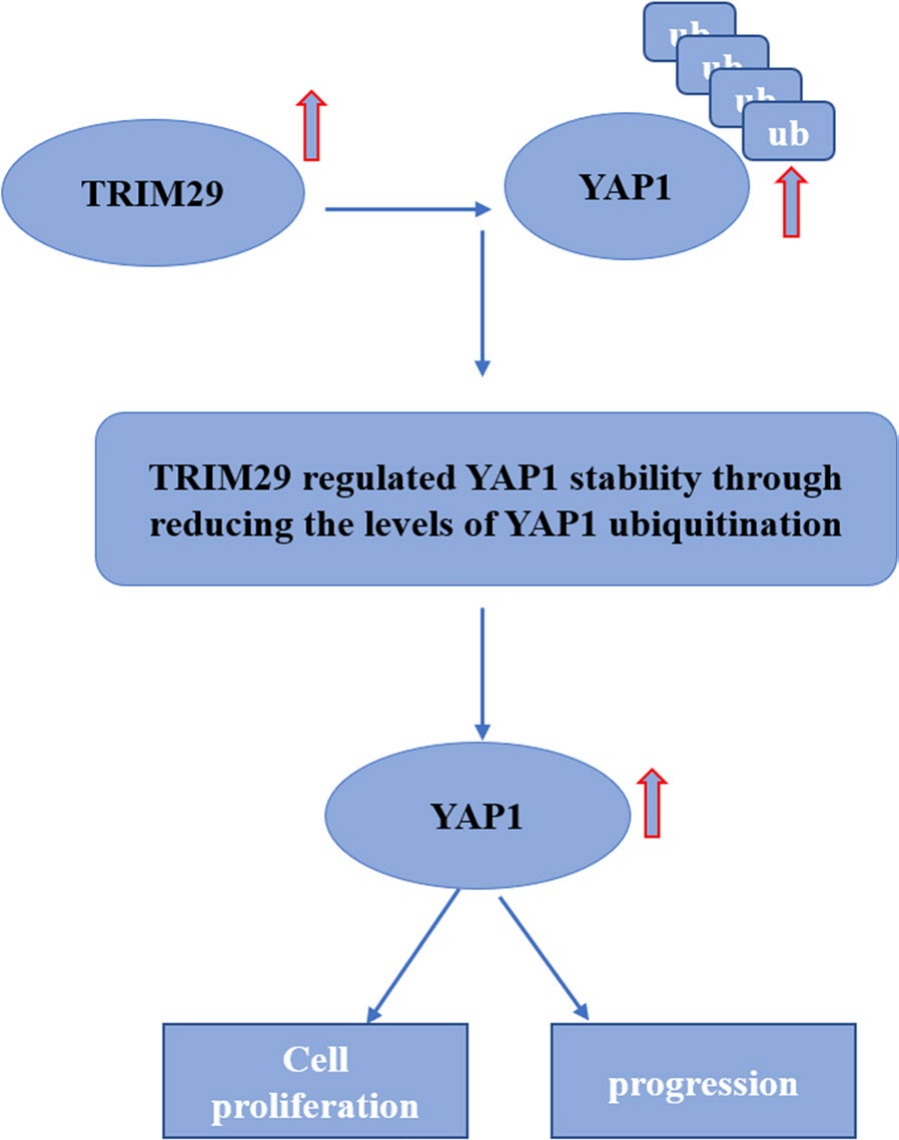
The schematic illustration of the potential molecular mechanism of TRIM29 as a key regulator in PC progression. TRIM29 promotes PC growth and progression via stabilizing Yes-associated protein 1

In conclusion, we provided evidence that TRIM29 is upregulated in pancreatic cancer tissues and is associated with PC progression. Moreover, TRIM29 promoted the growth of PC cells. More importantly, TRIM29-induced growth was dependent on YAP1 expression in PC cells. Our findings also demonstrated that TRIM29 directly interacts with YAP1, which then affects YAP1 ubiquitination and degradation (Fig. 7). Based on these findings, we conclude that TRIM29 is a candidate biomarker for PC diagnosis and a novel potential therapeutic target.

## Supplementary Information

**Additional file 1.** Cell Line Authentication of PANC-1.

**Additional file 2.** Cell Line Authentication of BxPC-3.

**Additional file 3.** Cell Line Authentication of AsPC-1.

## Data Availability

The datasets generated and analyzed during the current study are available from the corresponding author upon reasonable request.

## Abbreviations

PC: Pancreatic cancer
TRIM29: Tripartite motif-29
YAP1: Yes-associated protein 1
OS: Overall survival
qRT-PCR: Quantitative real-time PCR
IHC: Immunohistochemistry
Co-IP: Co-immunoprecipitation
ANOVA: One-way analysis of variance
GEPIA: Gene Expression Profiling Interactive Analysis
