# Supplementary material for: E3 ubiquitin ligase TRIM29 promotes pancreatic cancer growth and progression via stabilizing Yes-associated protein 1
Source: J Transl Med. 2021 Aug 5;19:332. doi: 10.1186/s12967-021-03007-w (PMC8340474; doi:10.1186/s12967-021-03007-w)
Supplement: Supplementary file 2 — Additional file 2. Cell Line Authentication of BxPC-3. [file 12967_2021_3007_MOESM2_ESM.pdf]

# Cell Line Authentication Service

## STR Profiling Report

**Sample From:** The second affiliated hospital of Nanchang university

**Sample Type:** Cell Line

**Testing Method:** STR Genotyping

**Report Time:** July 1, 2021


**Add:** Room 502, NO.1015 Longteng Rd , Songjiang District, Shanghai/ Tel:+86-021-33559491
Homepage: http://www.biowing.com.cn/ E-mail: market@biowing.com.cn

# COMPANY STATEMENT

1. THIS REPORT IS ONLY RESPONSIBLE FOR THE SAMPLES ANALYZED.
2. THE TESTING RESULTS AND THE ORGANIZATION NAME WILL NOT BE USED FOR ADVERTISEMENT, COMMERCIAL EXHIBITIONS, COMMERCIAL PERFORMANCE AND OTHER COMMERCIAL ACTIVITIES.
3. OBJECTIONS SHOULD BE RAISED WITHIN FIFTEEN DAYS AFTER THE RECEIPT OF THIS REPORT.
4. THE PAPER REPORT WITH CONTENT ALTERING, ADDING ARE INVALID.

**Testing Company:** Shanghai Biowing Applied Biotechnology Co. Ltd
**Address:** Room 502, NO.1015 Longteng Rd , Songjiang District, Shanghai
**Tel:** +86-021-33559491
**Contact:** Wenyao Zhang
**E-mail:** market@biowing.com.cn

# Cell Line Authentication – STR Profiling Report

## Sample code

Table 1. Sample Code

| Customer's code | Company Code |
|---|---|
| PANC-1 | 20200624-01 |

**Sample Number** :1

**Sample Type**: Cell line

**Testing Type**: STR

**Testing Method**:

DNA was extracted by a commercial kit from CORNING (AP-EMN-BL-GDNA-250G). The twenty STRs including Amelogenin locus were amplified by six multiplex PCR and separated on ABI 3730XL Genetic Analyzer. The signals were then analyzed by the software GeneMapper.

## Data Interpretation:

Cell lines were authenticated using Short Tandem Repeat (STR) analysis asdescribed in 2012 in ANSI Standard (ASN-0002) by the ATCC Standards Development Organization (SDO) and in Capes-Davis et al., Match criteria for human cell line authentication: Where do we draw the line? Int J Cancer.2013;132(11):2510-9.

# Test Results

## 1. STR profile

Table 2. STR and Amelogenin Genotyping Results of Cell line.

| Loci | Sample information | | | Cell Bank information | | |
|---|---|---|---|---|---|---|
| | Sample name：PANC-1 | | | Cell line name：PANC-1 | | |
| | **Allele1** | **Allele2** | **Allele3** | **Allele1** | **Allele2** | **Allele3** |
| D5S818 | 11 | 13 | | 11 | 13 | |
| D13S317 | 11 | 11 | | 11 | 11 | |
| D7S820 | 8 | 10 | | 8 | 10 | |
| D16S539 | 11 | 11 | | 11 | 11 | |
| VWA | 15 | 15 | | 15 | 15 | |
| TH01 | 7 | 8 | | 7 | 8 | |
| AMEL | X | X | | X | X | |
| TPOX | 8 | 11 | | 8 | 11 | |
| CSF1PO | 10 | 12 | | 10 | 12 | |
| D12S391 | 22 | 22 | | | | |
| FGA | 21 | 21 | | | | |
| D2S1338 | 23 | 24 | | | | |
| D21S11 | 28 | 28 | | | | |
| D18S51 | 12 | 12 | | | | |
| D8S1179 | 14 | 15 | | | | |
| D3S1358 | 17 | 17 | | | | |
| D6S1043 | 11 | 12 | | | | |
| PENTAE | 7 | 14 | | | | |
| D19S433 | 11 | 16 | | | | |
| PENTAD | 14 | 14 | | | | |
| D1S1656 | 12 | 14 | | | | |


Add: Room 502, NO.1015 Longteng Rd , Songjiang District, Shanghai/ Tel:+86-021-33559491
Homepage: http://www.biowing.com.cn/ E-mail: market@biowing.com.cn

## 2. database annotation

Figure 1. STR matching analysis

| EV | Cell No. | Cell name | Locus names | | | | | | | | |
|---|---|---|---|---|---|---|---|---|---|---|---|
| | | | D5S818 | D13S317 | D7S820 | D16S539 | VWA | TH01 | AM | TPOX | CSF1PO |
| | *Query (Your Cell)* | | *11,13* | *11,11* | *8,10* | *11,11* | *15,15* | *7,8* | *X,X* | *8,11* | *10,12* |
| 1.00(36/36) | 783 | PANC-1 | 11,13 | 11,11 | 8,10 | 11,11 | 15,15 | 7,8 | X,X | 8,11 | 10,12 |
| 1.00(36/36) | CRL-1469 | PANC-1 | 11,13 | 11,11 | 8,10 | 11,11 | 15,15 | 7,8 | X,X | 8,11 | 10,12 |

**Note:** The STR online match analysis of the test cell against DSMZ database, showing cell number (Cell No.) and cell name.

## 3. Authentication

☐ The submitted sample profile is human, but not a match for any profile in the DSMZ STR database.

☑ The submitted profile is exact match for the following human cell line(s) in the DSMZ STR database (8 core loci plus Amelogenin): **PANC-1**.

☐ The submitted profile is similar to the following DSMZ human cell line: /.

- **Note:** A cell line can be considered to be authenticated when 80% (exact match) of the alleles in its STR profile match profiles from tissue or other cell line samples from that donor or from database. Cell lines with between a 55% to 80% (similar) match require further profiling for investigation of relatedness.

Figure 2. STR profiles of sample cell line

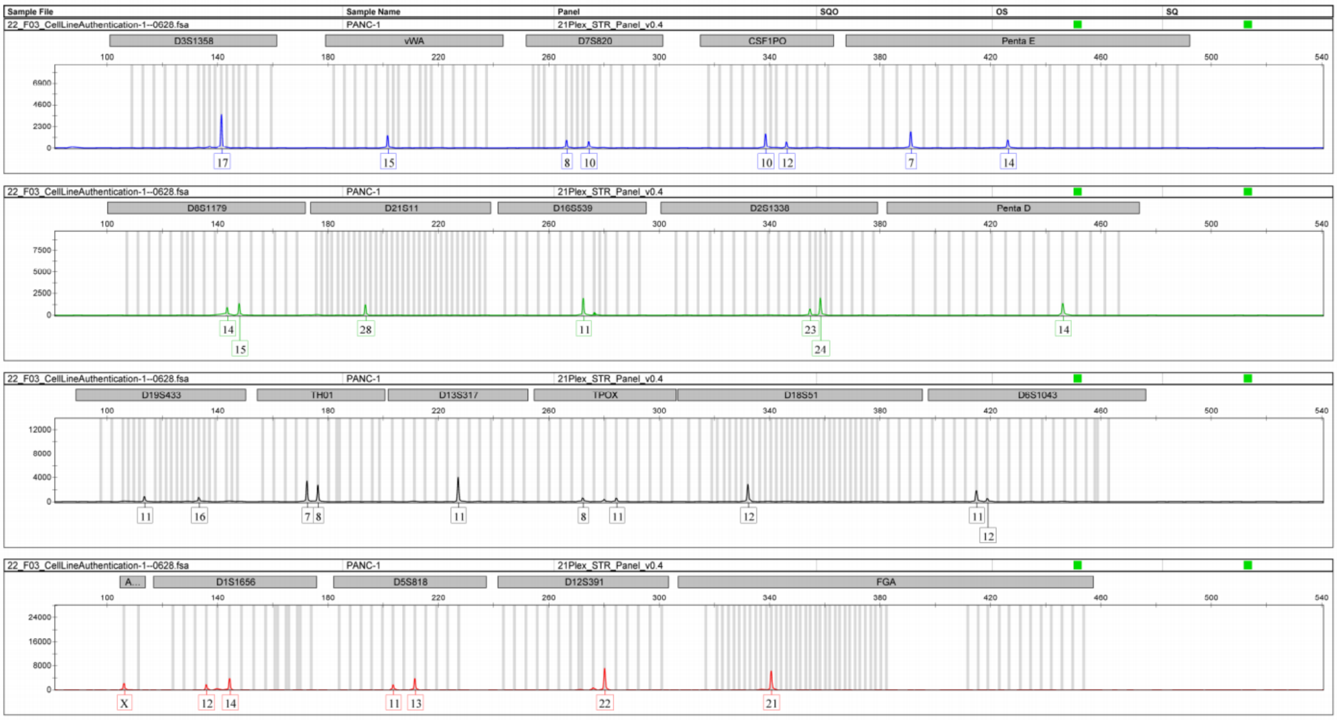



Add: Room 502, NO.1015 Longteng Rd , Songjiang District, Shanghai/ Tel:+86-021-33559491
Homepage: http://www.biowing.com.cn/ E-mail: market@biowing.com.cn

# Appendix

1. Genotyping Strategy and Site Distribution

Table S1. Experimental Strategy and Sites

| | **Strategy 1** | **Strategy 2** | **Strategy 3** | **Strategy 4** |
|---|---|---|---|---|
| 1 | D3S1358 | D8S1179 | D19S433 | AMEL |
| 2 | VWA | D21S11 | TH01 | D1S1656 |
| 3 | D7S820 | D16S539 | D13S317 | D5S818 |
| 4 | CSF1PO | D2S1338 | TPOX | D12S391 |
| 5 | PENTAE | PENTAD | D18S51 | FGA |
| 6 | | | D6S1043 | |

*The allele match algorithm compares the 8 core loci plus amelogenin only, even though alleles from all lociwill be reported when available.*

2. DSMZ tools was used to carry on the cell line comparison, which contains 2455 cell lines STR data from ATCC, DSMZ, JCRB ,ECACC， GNE and RIKEN databases. If the cell is not included in the above cell library, users need to compared with other databases.

**Technician:** Jianan Zhang

**Checked by:** Shangning Zhu

**Issued by**: Yang Bai

**Issue date:** July 1, 2021


Add: Room 502, NO.1015 Longteng Rd , Songjiang District, Shanghai/ Tel:+86-021-33559491
Homepage: http://www.biowing.com.cn/ E-mail: market@biowing.com.cn
