# Supplementary material for: E3 ubiquitin ligase TRIM29 promotes pancreatic cancer growth and progression via stabilizing Yes-associated protein 1
Source: J Transl Med. 2021 Aug 5;19:332. doi: 10.1186/s12967-021-03007-w (PMC8340474; doi:10.1186/s12967-021-03007-w)
Supplement: Supplementary file 3 — Additional file 3. Cell Line Authentication of AsPC-1. [file 12967_2021_3007_MOESM3_ESM.pdf]

Cell Line Authentication Service

STR Profiling Report

Sample From: The second affiliated hospital of Nanchang university

Sample Type: Cell Line

Testing Method: STR Genotyping

Report Time: June 17, 2021

Add: Room 502, NO.1015 Longteng Rd , Songjiang District, Shanghai/ Tel:+86-021-33559491
Homepage: http://www.biowing.com.cn/ E-mail: market@biowing.com.cn

COMPANY STATEMENT

1. THIS REPORT IS ONLY RESPONSIBLE FOR THE SAMPLES ANALYZED.
2. THE TESTING RESULTS AND THE ORGANIZATION NAME WILL NOT BE USED FOR ADVERTISEMENT, COMMERCIAL EXHIBITIONS, COMMERCIAL PERFORMANCE AND OTHER COMMERCIAL ACTIVITIES.
3. OBJECTIONS SHOULD BE RAISED WITHIN FIFTEEN DAYS AFTER THE RECEIPT OF THIS REPORT.
4. THE PAPER REPORT WITH CONTENT ALTERING, ADDING ARE INVALID.

Testing Company: Shanghai Biowing Applied Biotechnology Co. Ltd
Address: Room 502, NO.1015 Longteng Rd , Songjiang District, Shanghai
Tel: +86-021-33559491
Contact: Wenyao Zhang
E-mail: market@biowing.com.cn

Cell Line Authentication – STR Profiling Report

Sample code

Table 1. Sample Code

Customer's code	Company Code
ASPC-1	20200614-02

Sample Number :1

Sample Type: Cell line

Testing Type: STR

Testing Method:

DNA was extracted by a commercial kit from CORNING (AP-EMN-BL-GDNA-250G). The twenty STRs including Amelogenin locus were amplified by six multiplex PCR and separated on ABI 3730XL Genetic Analyzer. The signals were then analyzed by the software GeneMapper.

Data Interpretation:

Cell lines were authenticated using Short Tandem Repeat (STR) analysis asdescribed in 2012 in ANSI Standard (ASN-0002) by the ATCC Standards Development Organization (SDO) and in Capes-Davis et al., Match criteria for human cell line authentication: Where do we draw the line? Int J Cancer.2013;132(11):2510-9.

Add: Room 502, NO.1015 Longteng Rd , Songjiang District, Shanghai/ Tel:+86-021-33559491
Homepage: http://www.biowing.com.cn/ E-mail: market@biowing.com.cn

Test Results

1. STR profile

Table 2. STR and Amelogenin Genotyping Results of Cell line.

Loci	**Sample information**			**Cell Bank information**		
	Sample name: ASPC-1			Cell line name: AsPC-1		
	**Allele1**	**Allele2**	**Allele3**	**Allele1**	**Allele2**	**Allele3**
D5S818	12	12		12	12	
D13S317	9	12		9	12	
D7S820	12	13		12	13	
D16S539	11	11		11	11	
VWA	17	17		17	17	
TH01	7	9.3		7	9.3	
AMEL	X	X		X	X	
TPOX	8	10		8	10	
CSF1PO	10	13		10	13	
D12S391	19	19				
FGA	24	24				
D2S1338	22	23				
D21S11	28	30				
D18S51	18	18				
D8S1179	13	15				
D3S1358	16	16				
D6S1043	11	11				
PENTAE	5	12				
D19S433	14	14				
PENTAD	9	12				
D1S1656	12	18.3				

Add: Room 502, NO.1015 Longteng Rd , Songjiang District, Shanghai/ Tel:+86-021-33559491
Homepage: http://www.biowing.com.cn/ E-mail: market@biowing.com.cn

2. database annotation

Figure 1. STR matching analysis

EV	Cell No.	Cell name	Locus names								
			D5S818	D13S317	D7S820	D16S539	VWA	TH01	AM	TPOX	CSF1PO
	Query (Your Cell)		*12,12*	*9,12*	*12,13*	*11,11*	*17,17*	*7,9.3*	*X,X*	*8,10*	*10,13*
1.00(36/36)	CRL-1682	AsPC-1	12,12	9,12	12,13	11,11	17,17	7,9.3	X,X	8,10	10,13

Note: The STR online match analysis of the test cell against DSMZ database, showing cell number (Cell No.) and cell name.

3. Authentication

☐ The submitted sample profile is human, but not a match for any profile in the DSMZ STR database.

☑ The submitted profile is exact match for the following human cell line(s) in the DSMZ STR database (8 core loci plus Amelogenin): **AsPC-1**.

☐ The submitted profile is similar to the following DSMZ human cell line: /.

- **Note:** A cell line can be considered to be authenticated when 80% (exact match) of the alleles in its STR profile match profiles from tissue or other cell line samples from that donor or from database. Cell lines with between a 55% to 80% (similar) match require further profiling for investigation of relatedness.

Figure 2. STR profiles of sample cell line

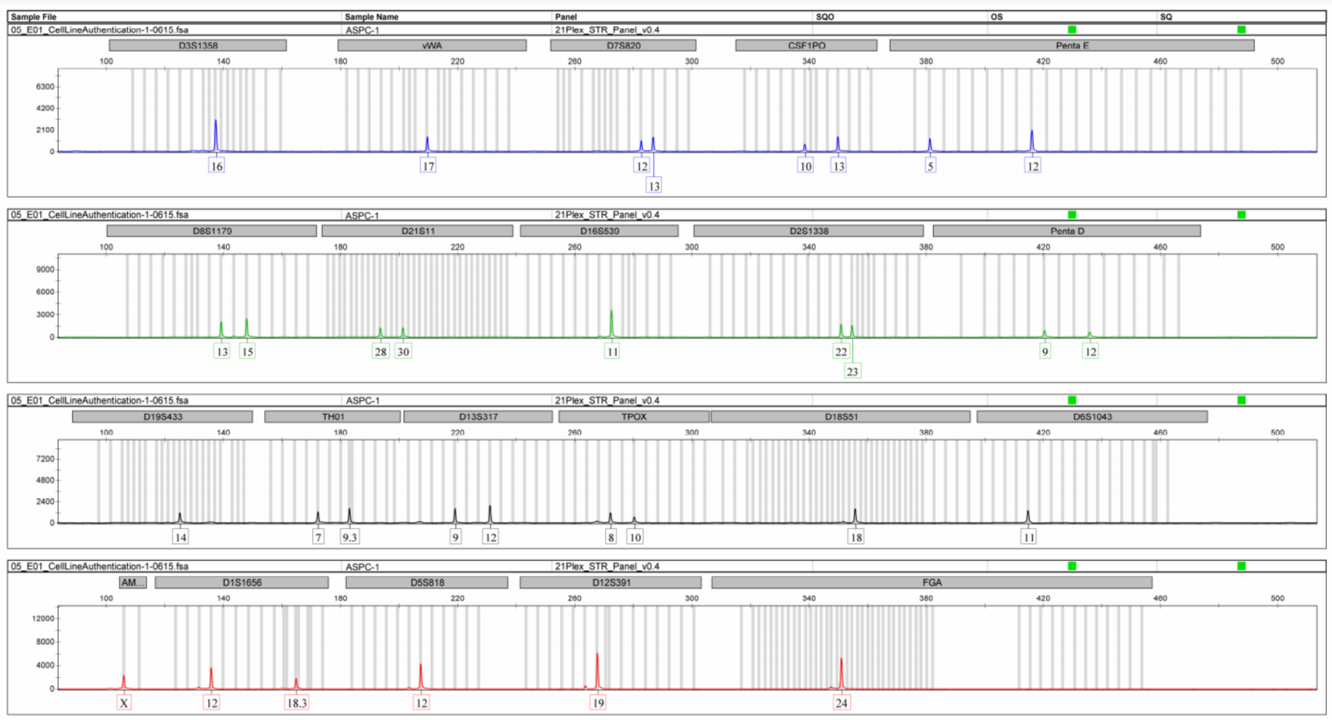

Add: Room 502, NO.1015 Longteng Rd , Songjiang District, Shanghai/ Tel:+86-021-33559491
Homepage: http://www.biowing.com.cn/ E-mail: market@biowing.com.cn

Appendix

1. Genotyping Strategy and Site Distribution

Table S1. Experimental Strategy and Sites

	Strategy 1	Strategy 2	Strategy 3	Strategy 4
1	D3S1358	D8S1179	D19S433	AMEL
2	VWA	D21S11	TH01	D1S1656
3	D7S820	D16S539	D13S317	D5S818
4	CSF1PO	D2S1338	TPOX	D12S391
5	PENTAE	PENTAD	D18S51	FGA
6			D6S1043	

The allele match algorithm compares the 8 core loci plus amelogenin only, even though alleles from all lociwill be reported when available.

2. DSMZ tools was used to carry on the cell line comparison, which contains 2455 cell lines STR data from ATCC, DSMZ, JCRB ,ECACC，GNE and RIKEN databases. If the cell is not included in the above cell library, users need to compared with other databases.

Technician: Jianan Zhang

Checked by: Shangning Zhu

Issued by: Yang Bai

Issue date: June 17, 2021

Add: Room 502, NO.1015 Longteng Rd , Songjiang District, Shanghai/ Tel:+86-021-33559491
Homepage: http://www.biowing.com.cn/ E-mail: market@biowing.com.cn
